# Rapid Emergence and Epidemiologic Characteristics of the SARS-CoV-2 B.1.526 Variant — New York City, New York, January 1–April 5, 2021

**DOI:** 10.15585/mmwr.mm7019e1

**Published:** 2021-05-14

**Authors:** Corinne N. Thompson, Scott Hughes, Stephanie Ngai, Jennifer Baumgartner, Jade C. Wang, Emily McGibbon, Katelynn Devinney, Elizabeth Luoma, Daniel Bertolino, Christina Hwang, Kelsey Kepler, Cybill Del Castillo, Melissa Hopkins, Henry Lee, Andrea K. DeVito, Jennifer L. Rakeman, PhD, Anne D. Fine

**Affiliations:** ^1^New York City Department of Health and Mental Hygiene, Long Island City, New York; ^2^Pandemic Response Laboratory, New York, New York; ^3^Department of Genetics, Harvard Medical School, Boston, Massachusetts.

Recent studies have documented the emergence and rapid growth of B.1.526, a novel variant of interest (VOI) of SARS-CoV-2, the virus that causes COVID-19, in the New York City (NYC) area after its identification in NYC in November 2020 (*1*–*3*). Two predominant subclades within the B.1.526 lineage have been identified, one containing the E484K mutation in the receptor-binding domain (*1*,*2*), which attenuates in vitro neutralization by multiple SARS-CoV-2 antibodies and is present in variants of concern (VOCs) first identified in South Africa (B.1.351) (*4*) and Brazil (P.1).* The NYC Department of Health and Mental Hygiene (DOHMH) analyzed laboratory and epidemiologic data to characterize cases of B.1.526 infection, including illness severity, transmission to close contacts, rates of possible reinfection, and laboratory-diagnosed breakthrough infections among vaccinated persons. Preliminary data suggest that the B.1.526 variant does not lead to more severe disease and is not associated with increased risk for infection after vaccination (breakthrough infection) or reinfection. Because relatively few specimens were sequenced over the study period, the statistical power might have been insufficient to detect modest differences in rates of uncommon outcomes such as breakthrough infection or reinfection. Collection of timely viral genomic data for a larger proportion of citywide cases and rapid integration with population-based surveillance data would enable improved understanding of the impact of emerging SARS-CoV-2 variants and specific mutations to help guide public health intervention efforts.

SARS-CoV-2 specimens were sequenced at the Public Health Laboratory (PHL) or the Pandemic Response Laboratory (PRL). During January 1–April 5, 2021, PHL received specimens primarily from NYC residents at nine COVID Express laboratories. All nucleic acid amplification test (NAAT)-positive SARS-CoV-2 specimens with a cycle threshold (Ct) value <32 underwent whole genome sequencing (WGS) (Scott Hughes, PhD, NYC PHL, personal communication, April 2021). At PRL, specimens collected at approximately 190 outpatient facilities were randomly selected, and those with a Ct value ≤30 were sequenced (*5*,*6*). Characteristics of persons with sequenced viruses were compared with those of NYC residents with COVID-19 diagnoses during the same period to evaluate representativeness. Records of persons with sequenced viruses were matched to the DOHMH COVID-19 surveillance Citywide Immunization and Vital Registry databases.

Persons infected with B.1.526 were compared with persons infected with variants that were not classified as VOIs or VOCs (i.e., non-VOI/VOC infections).^†^ Persons infected with B.1.526 were also compared with those infected with B.1.1.7 because of the recent increase in B.1.1.7 cases in NYC and the documented increased transmissibility (*7*) and illness severity associated with this variant (*8*). To evaluate trends in socioeconomic status, neighborhood-level poverty was calculated as the percentage of residents in a ZIP code with household incomes <100% of the federal poverty level, per the American Community Survey 2014–2018. A case of possible reinfection was defined as an infection in a person with a sequenced specimen collected ≥90 days after a positive SARS-CoV-2 antigen or NAAT result. Breakthrough infections among partially vaccinated persons were defined as infections in persons with a sequenced specimen collected ≥14 days after the first vaccine dose and <14 days after the second dose (for mRNA vaccines). Breakthrough infections among fully vaccinated persons were defined as infections in persons with a sequenced specimen collected ≥14 days after either a second mRNA vaccine dose or a single dose viral vector vaccine. Comparisons across categorical characteristics were made using the chi-square or Fisher’s exact test; continuous variables were compared using the Kruskal-Wallis test (SAS Enterprise Guide, version 7.1).

WGS was completed on 9,765 SARS-CoV-2 specimens, including 1,186 (12%) sequenced at PHL and 8,579 (88%) at PRL, representing 3.1% of NAAT-positive cases identified citywide during January 1–April 5, 2021. The number of specimens undergoing WGS at these laboratories increased over time (Figure), representing 7.7% of all NAAT-positive specimens by the week ending April 5. The B.1.1.7 variant was identified in 1,815 (19%) specimens. Among 3,679 (38%) B.1.526 variant viruses identified, 2,050 (56%) carried the E484K mutation. The proportion of B.1.526 viruses identified increased from 3% in mid-January to 34% by February 22 (Figure) and stabilized at 35%‒45% weekly beginning March 8. The proportion of B.1.526 variants with the E484K mutation increased more quickly and as of April 5 represented 25% of all sequenced SARS-CoV-2 viruses, compared with 16% of B.1.526 variants without the E484K mutation. The proportion of B.1.1.7 viruses increased recently, reaching 36% by April 5. Other VOIs/VOCs were found among 253 specimens and were removed from additional analyses (B.1.427/B.1.429 variant in 166 specimens [viruses of B.1.427 or B.1.429 lineage, including those reported as B.1.427/B.1.429, without further differentiation]; P.1 variant in 50; B.1.525 variant in 20; B.1.351 variant in 12; and P.2 variant in 5). 

**FIGURE:**
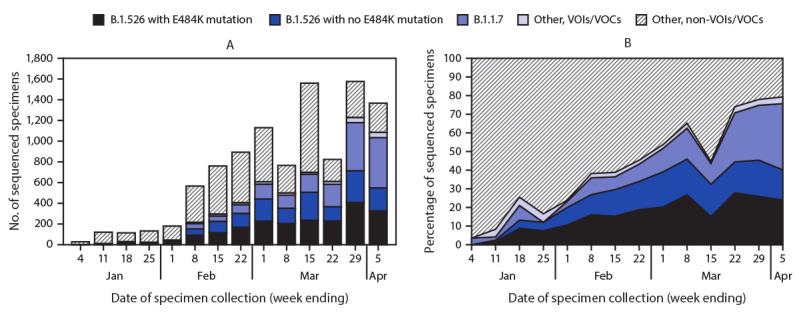
Number of specimens undergoing whole genome sequencing* (A) and percentage of specimens with B1.526 variant with or without E484K mutation, B.1.1.7 variant, and other variants of concern or interest (B), by week of specimen collection — New York City, New York, January 1–April 5, 2021 **Abbreviations:** VOC = variant of concern; VOI = variant of interest. * Whole genome sequencing of specimens (collection date during January 1–April 5, 2021) from New York City residents was performed at the Public Health Laboratory or the Pandemic Response Laboratory.

The geographic distribution of persons with viruses sequenced at PHL or PRL was similar to that of persons with positive SARS-CoV-2 NAAT tests citywide; however, these persons were more frequently aged <45 years (67% versus 60% citywide), residents of neighborhoods with high poverty or very high poverty (45% versus 40%), or Black/African American (19% versus 16%) or Hispanic/Latino (35% versus 28%). A lower percentage of these persons were hospitalized (4% versus 9%) and died (0.5% versus 1.5%).

Among 3,679 persons infected with the B.1.526 variant, the median age was 35 years (Table 1). Compared with persons with non-VOI/VOC infections, those with B.1.526 infections were significantly more likely to live in the Bronx or in neighborhoods with high or very high poverty or to identify as Black/African American. Among persons with B.1.526 infections, 2,618 (71%) were symptomatic, 104 (4.3%) were hospitalized, and 11 (0.5%) died; these proportions are similar to or lower than those in persons with non-VOI/VOC infections (71%, 4.1%, and 0.7%, respectively). However, persons infected with the B.1.1.7 variant were more likely to be hospitalized (5.8%) than were persons with non-VOI/VOC infections (4.1%) (p = 0.04) (Table 2).

**TABLE 1:** Number and percentage of SARS-CoV-2 variants* identified in specimens from New York City residents, by characteristics of residents — New York City, New York, January 1–April 5, 2021

Characteristic	Sequence result, no. (column %)			p-value^† ^(B.1.526 vs. other)
	B.1.526	B.1.1.7	Other (non-VOI/VOC)	
**Total^§^**	**3,679**	**1,815**	**4,271**	**—**
B.1.526 with E484K mutation	2,050 (55.7)	NA	NA	NA
**Median age, yrs (IQR)**	35 (23–50)	34 (22–48)	35 (23–51)	0.13
**Age group, yrs**				
0–17	626 (17.0)	318 (17.5)	721 (16.9)	0.04
18–44	1,857 (50.5)	954 (52.6)	2,071 (48.5)	
45–64	954 (25.9)	437 (24.1)	1,133 (26.5)	
65–74	154 (4.2)	74 (4.1)	238 (5.6)	
≥75	87 (2.4)	32 (1.8)	108 (2.5)	
**Sex**				
Male	1,671 (45.4)	818 (45.1)	2,056 (48.1)	0.02
Female	2,003 (54.4)	996 (54.9)	2,211 (51.8)	
**Race/Ethnicity^¶^**				
Hispanic	1,325 (43.0)	556 (38.2)	1,495 (42.3)	<0.001
Asian or Pacific Islander	413 (13.4)	169 (11.6)	496 (14.0)	
Black/African American	753 (24.4)	352 (24.2)	722 (20.4)	
White	534 (17.3)	351 (24.1)	757 (21.4)	
Other	59 (1.9)	26 (1.8)	66 (1.9)	
**Borough of residence**				
Bronx	870 (23.6)	256 (14.1)	790 (18.5)	<0.001
Brooklyn	945 (25.7)	552 (30.4)	1,068 (25.0)	
Manhattan	529 (14.4)	214 (11.8)	658 (15.4)	
Queens	1,124 (30.6)	584 (32.2)	1,465 (34.3)	
Staten Island	211 (5.7)	209 (11.5)	290 (6.8)	
Neighborhood poverty**				
Low (<10%)	386 (10.5)	304 (16.7)	583 (13.7)	<0.001
Medium (10%–19.9%)	1,401 (38.1)	721 (39.7)	1,715 (40.2)	
High (20%–29.9%)	1,128 (30.7)	553 (30.5)	1,215 (28.4)	
Very high (≥30%)	682 (18.5)	203 (11.2)	675 (15.8)	
**Clinical history**				
Symptomatic^††^	2,618 (71.2)	1,247 (68.7)	3,010 (70.5)	0.51
Possible reinfection^§§^	19 (0.5)	8 (0.4)	17 (0.4)	0.43
Ever had a positive serology result before specimen collection	38 (1.0)	13 (0.7)	37 (0.9)	0.44
**Vaccination history^¶¶^**				
No recorded dose	3,609 (98.1)	1,777 (97.9)	4,205 (98.5)	0.34
Partially vaccinated	59 (1.6)	31 (1.7)	52 (1.2)	
Fully vaccinated	11 (0.3)	7 (0.4)	14 (0.3)	

**Abbreviations:** IQR = interquartile range; NA = not applicable; Pangolin = Phylogenetic Assignment of Named Global Outbreak Lineages; VOC = variant of concern; VOI = variant of interest.

* Classified by Pangolin (https://pangolin.cog-uk.io/) identification of lineage as B.1.526, B.1.1.7, or other lineages that were not VOCs or VOIs. Whole genome sequencing of specimens (collected during January 1–April 5, 2021) was performed at the Public Health Laboratory or the Pandemic Response Laboratory.

^†^ p-values from chi-square test, Fisher’s exact test, or Kruskal-Wallis test as indicated, comparing persons with B.1.526 to persons with other non-VOI/VOC sequences.

^§^ This total does not include other VOCs/VOIs (N = 253): B.1.427/B.1.429 variant (n = 166), P.1 variant (n = 50), B.1.525 variant (n = 20), B.1.351 variant (n = 12), and P.2 variant (n = 5).

^¶^ Denominators are among persons with known race/ethnicity; 1,691 persons had missing race/ethnicity (595 persons with variant B.1.526, 361 persons with variant B.1.1.7, and 735 persons with other VOCs/VOIs). All persons who identified as Hispanic/Latino, regardless of race, are classified as such.

** Neighborhood-level poverty was defined as the percentage of residents in a ZIP code tabulation area with household incomes of <100% of the federal poverty level, per the American Community Survey 2014–2018.

^††^ Having at least two of the following: fever, chills, rigors, myalgia, headache, sore throat, nausea or vomiting, diarrhea, fatigue, congestion, or runny nose; or any one of the following: cough, shortness of breath, difficulty breathing, new olfactory disorder, or new taste disorder. https://wwwn.cdc.gov/nndss/conditions/coronavirus-disease-2019-covid-19/case-definition/2020/08/05/

**^§§^** An infection in a person with a sequenced specimen collected ≥90 days after collection of a specimen with a positive SARS-CoV-2 antigen or nucleic acid amplification test result.

^¶¶^ Partially vaccinated cases were defined as infections in persons with a sequenced specimen collected ≥14 days after the first vaccine dose and <14 days after the second dose (for mRNA vaccines). Fully vaccinated cases were defined as infections in persons with a sequenced specimen collected ≥14 days after a second mRNA vaccine dose or a single-dose viral vector vaccine.

**TABLE 2:** Number and percentage of SARS-CoV-2 variants* identified in specimens from New York City residents and number of hospitalizations, deaths, transmission to contacts, and clustering in buildings or households — New York City, New York, January 1–March 22, 2021^†^

Characteristic	Sequence result, no. (column %)			p-value^§^ (B.1.526 vs. other)
	B.1.526	B.1.1.7	Other (non-VOI/VOC)	
**Total**	**2,416**	**865**	**3,640**	**—**
Hospitalized within 14 days of specimen collection	104 (4.3)	50 (5.8)	151 (4.1)	0.77
Death within 60 days of specimen collection	11 (0.5)	4 (0.5)	27 (0.7)	0.17
Persons with COVID-19 with any known contacts^¶^	801/2,303 (34.8)	254/791 (32.1)	1,196/3,564 (33.6)	0.33
At least one contact had COVID-19**	359/801 (44.8)	99/254 (39.0)	520/1,196 (43.5)	0.55
Persons with COVID-19 with any known household contacts^¶^	735/2,303 (31.9)	240/791 (30.3)	1,102/3,654 (30.9)	0.42
At least one household contact had COVID-19**	327/735 (44.5)	97/240 (40.4)	464/1,102 (42.1)	0.31
COVID-19 cases associated with a building or household cluster^††^	1,482/2,199 (67.4)	474/769 (61.6)	2,241/3,386 (66.2)	0.35

**Abbreviations:** Pangolin = Phylogenetic Assignment of Named Global Outbreak Lineages; VOC = variant of concern; VOI = variant of interest.

* Classified by Pangolin (https://pangolin.cog-uk.io/) identification of lineage as B.1.526, B.1.1.7, or other non-VOC/VOI lineages. Whole genome sequencing of specimens (collected during January 1–April 5, 2021) was performed at the Public Health Laboratory or the Pandemic Response Laboratory.

^†^ Persons with specimens collected March 23–April 5 were excluded from severity and transmission metrics because outcomes such as hospitalization, death, or transmission as well as diagnosis of contacts typically take weeks to occur and would not have been reported in time to be included.

^§^ p-values from chi-square test, Fisher’s exact test, or Kruskal-Wallis test as indicated, comparing persons with B.1.526 sequences and persons with other non-VOI/VOC sequences.

^¶^ Contacts were reported by persons with COVID-19 and include those reported with a last name, first name, and date of birth.

** Among persons with known COVID-19; contacts are considered to be persons with known COVID-19 if they were infected with SARS-COV-2 and had a diagnosis date or an onset date within the 14 days after their exposure; the denominator is different because of the exclusion of residents of congregate settings.

^††^ A building cluster was defined as three or more confirmed cases with positive antigen test results within 21 days in the same building, and a household cluster was defined as two or more confirmed cases with positive antigen test results within 21 days in the same household, based on shared last name or unit number (or both); the denominator is different because only persons with COVID-19 with valid noncongregate residential addresses are included.

Possible reinfections were rare overall (0.5%), and the prevalence was similar among all persons with sequenced specimens (Table 1). No difference in rates of possible reinfection was found between persons infected with B.1.1.7 variants and those infected with B.1.526 variants with or without the E484K mutation (Supplementary Figure, https://stacks.cdc.gov/view/cdc/105634). The proportion of persons with a previous positive serology test result was 0.9% overall and similar among patients infected with all lineages (Table 1). Among persons infected with the B.1.526 variant carrying the E484K mutation, previous seropositivity was slightly more common (1.3%) than that among persons infected with the B.1.526 variant without the E484K mutation (0.7%), with the B.1.1.7 variant (0.7%), and with other non-VOI/VOC infections (0.9%); however, the difference was not significant (p = 0.23).

Among 32 fully vaccinated persons with sequenced viruses, eight (25%) were identified who were infected with the B.1.526 variant carrying the E484K mutation, three (9%) with the B.1.526 variant without the E484K mutation, seven (22%) with the B.1.1.7 variant, and 14 (44%) with non-VOI/VOC infections. No major differences between persons with B.1.526 and non-VOI/VOC infections were found in the secondary COVID-19 attack rate among household or community members identified as close contacts (Table 2).

## Discussion

The B.1.526 SARS-CoV-2 lineage was identified in NYC in November 2020 (*1*), and its prevalence has increased sharply since mid-January 2021. By April 5, the B.1.526 variant accounted for 40% of all viruses sequenced by two major laboratories from a relatively representative sample of NYC COVID-19 cases. Approximately one half of the B.1.526 variants identified were found to have the E484K mutation, which has been shown to attenuate antibody neutralization in vitro (*3*). Although the proportional increase in B.1.526 infections suggests that this variant might be more transmissible than other SARS-CoV-2 variants, the secondary attack rate was not higher. Compared with persons infected with non-VOI/VOC viruses, B.1.526 appears to be slightly more prevalent in populations that have experienced disproportionate levels of COVID-19–associated morbidity and mortality (*9*) and that have lower vaccination rates than higher income NYC populations (*10*).

These preliminary data suggest that the SARS-CoV-2 B.1.526 variant does not cause more severe disease. In NYC, evidence does not indicate a higher reinfection rate among persons infected with B.1.526 viruses carrying the E484K mutation compared with those with infections without the mutation, although this might reflect incomplete case ascertainment during early 2020 because of limited testing capacity. Whereas a slightly larger proportion of persons infected with the B.1.526 variant carrying the E484K mutation had a previous positive antibody test than those infected with B.1.526 without the mutation, the difference is not significant, and data are insufficient to conclude that there is an increased risk for reinfection. Laboratory studies of B.1.526 variants carrying the E484K mutation showed that vaccine-induced antibodies against this virus had decreased neutralizing activity and that certain monoclonal antibodies had impaired activity^§^ (*3*). Additional evaluations in human populations are required to assess immune evasion of B.1.526.

The findings in this report are subject to at least four limitations. First, the majority of documented B.1.526 infections are recent, and because of lags in the confirmation of hospitalizations and deaths and incomplete ascertainment of previous infection or vaccination, drawing conclusions regarding severity of infection, risk for reinfection, vaccine breakthrough, or secondary attack rate is challenging. Second, infections in persons with sequenced viruses represent a small proportion of diagnosed cases; the lower rate of hospitalizations and deaths in this population might limit the ability to detect a difference in severe outcomes associated with the B.1.526 variant. However, the lack of evidence for increased severity of the B.1.526 variant contrasts with the significantly higher hospitalization rate that was observed among persons with B.1.1.7 infections using these methods, which is consistent with evidence that this variant has increased virulence (*8*). Third, persons with sequenced viruses might differ from those with nonsequenced viruses. Finally, the population with the highest prevalence of B.1.526 infection in NYC has lower vaccination rates, limiting the ability to discern an increased risk for vaccine breakthrough (*10*).

Although the SARS-CoV-2 B.1.526 variant emerged rapidly in NYC, early evidence suggests that this variant, even with the E484K mutation, does not lead to more severe disease and is not associated with increased risk for breakthrough infection or reinfection compared with other sequenced SARS-CoV-2 viruses. The number of persons with reinfection or breakthrough infection whose specimens underwent WGS is low, limiting the statistical power to detect modest increases in immune escape that could have a substantial impact on public health. Improved capacity for genomic surveillance, establishment of automated and efficient exchange of WGS data, and integration with population-based clinical and epidemiologic data would enable the rapid characterization of emerging SARS-CoV-2 variants, which could guide public health policies related to reopening, prevention strategies, identifying areas for vaccination, and guiding future vaccine development.

**Summary**

**What is already known about this topic?**

B.1.526 emerged in November 2020 as a SARS-CoV-2 variant of interest in New York City (NYC). The presence of the E484K mutation is concerning because it has been shown to attenuate antibody neutralization in vitro.

**What is added by this report?**

The NYC Department of Health and Mental Hygiene analyzed laboratory and epidemiologic data to characterize cases of B.1.526 infection and the associated potential for breakthrough infection and reinfection. Preliminary evidence suggests that, to date, B.1.526 does not lead to more severe disease or increased risk for infection after vaccination.

**What are the implications for public health practice?**

Rapid integration of whole genome sequencing and population-based surveillance data is critical to characterizing new SARS-CoV-2 variants.

## Footnotes

*
https://www.medrxiv.org/content/10.1101/2021.02.26.21252554v1

†
https://www.cdc.gov/coronavirus/2019-ncov/cases-updates/variant-surveillance/variant-info.html

§
https://www.biorxiv.org/content/10.1101/2021.03.24.436620v1
